# A hypoxia-dependent upregulation of hypoxia-inducible factor-1 by nuclear factor-*κ*B promotes gastric tumour growth and angiogenesis

**DOI:** 10.1038/sj.bjc.6606020

**Published:** 2010-11-30

**Authors:** S Y Nam, Y S Ko, J Jung, J Yoon, Y H Kim, Y J Choi, J W Park, M S Chang, W H Kim, B L Lee

**Affiliations:** 1 Radiation Health Research Institute, Korea Hydro & Nuclear Power Co., Ltd., Seoul 132-703, Korea; 2 Department of Anatomy, Seoul National University College of Medicine, 28 Yongon-dong, Chongno-gu, Seoul 110-799, Korea; 3 Inha University College of Medicine, Incheon 402-751, Korea; 4 Department of Pharmacology, Seoul National University College of Medicine, Seoul 110-799, Korea; 5 Ischemic/Hypoxic Disease Institute Medical Research Center, Seoul National University College of Medicine, Seoul 110-799, Korea; 6 Department of Pathology, Seoul National University College of Medicine, Seoul 110-799, Korea; 7 Cancer Research Institute, Seoul National University College of Medicine, Seoul 110-799, Korea

**Keywords:** HIF-1*α*, NF-*κ*B, gastric cancer, tumour growth, angiogenesis, hypoxia

## Abstract

**Background:**

The underlying mechanisms involved in the activation of hypoxia-inducible factor-1 (HIF-1) in gastric cancer remain unclear. As nuclear factor-*κ*B (NF-*κ*B) as well as HIF-1 have been implicated in angiogenesis of various cancers, we investigated their relationship in gastric cancer.

**Methods:**

Nuclear expressions of HIF-1*α* and NF-*κ*B/RelA were assessed in 251 human gastric carcinoma specimens by immunohistochemical tissue array analysis. Stable human gastric cancer cells, infected with a retroviral vector containing super-suppressive mutant form of I*κ*B*α* (I*κ*B*α*M), were used for animal studies as well as cell culture experiments. Xenografted tumours were measured and I*κ*B*α*M effects on angiogenesis and HIF-1*α* activation were assessed by immunohistochemistry, western blotting, luciferase reporter assay, and semiquantitative reverse transcription–polymerase chain reaction. In addition, NF-*κ*B effects on the HIF-1*α* degradation and synthesis were examined.

**Results:**

Hypoxia-inducible factor-1*α* activation positively correlated with RelA activation in clinical gastric cancer samples (*P*<0.001). The I*κ*B*α*M overexpression suppressed tumour growth, microvessel density, and HIF-1*α* activation in xenografted tumours. Cell culture experiments showed that hypoxia-induced HIF-1*α* expression was reduced by NF-*κ*B inhibition under hypoxic conditions at the translational level.

**Conclusion:**

The hypoxia-dependent activation of the NF-*κ*B/HIF-1*α*/VEGF pathway contributes, at least in part, to gastric cancer promotion via enhancement of angiogenesis.

Hypoxia commonly develops within solid tumours and stimulates the expression of several genes responsible for tumour cell survival, proliferation, and angiogenesis (Royds *et al*, 1998; Giatromanolaki and Harris, 2001). As hypoxia induces the development of a clinically aggressive tumour phenotype as well as resistance to chemotherapy and radiation (Royds *et al*, 1998; Höckel and Vaupel, 2001), many elements of the hypoxia-response pathway are good candidates for therapeutic targets. However, the mechanism that relates hypoxia to these tumour characteristics has remained unclear.

Hypoxia-inducible factor-1 (HIF-1) is a key transcription factor that regulates blood vessel formation by affecting the expression of vascular endothelial growth factor (VEGF) (Forsythe *et al*, 1996; Hong *et al*, 2004). It has a key role in cellular responses to hypoxia in both normal and malignant cells (Iyer *et al*, 1998; Semenza, 2000). As HIF-1 is a heterodimeric transcription factor composed of oxygen-dependent HIF-1*α* and constitutively expressed HIF-1*β* subunits, HIF-1 transcriptional activity is largely determined by regulated expression of the HIF-1*α* subunit (Semenza, 2001). Although HIF-1*α* is stabilised under hypoxic conditions because of its oxygen-dependent degradation domain that binds with the von-Hippel-Lindau protein (pVHL)-containing complex, non-hypoxic activation of HIF-1*α* by cytokines, oncogenes, and reactive oxygen species has also been reported (Dery *et al*, 2005). However, the detailed molecular mechanism underlying HIF-1 activation could be different according to cell type (Li *et al*, 2005).

Nuclear factor-*κ*B (NF-*κ*B) is a critical transcription factor in various cancers that regulates genes associated with a variety of cellular functions, such as cell survival, proliferation, angiogenesis, and cancer metastasis (Guttridge *et al*, 1999; Baldwin, 2001a, 2001b; Karashima *et al*, 2003; Meir *et al*, 2007). Nuclear factor-*κ*B is normally sequestered in the cytoplasm of nonstimulated cells by a family of inhibitory proteins, I*κ*Bs, which bind to NF-*κ*B and mask the nuclear localisation signal domain of NF-*κ*B. Once cells are exposed to extracellular stimuli, I*κ*Bs become phosphorylated, ubiquitinated, and degraded by the proteasomes, leading to NF-*κ*B activation (Gilmore, 1999).

Both HIF-1 and NF-*κ*B are involved in cancer progression (Rayet and Gélinas, 1999; Hare *et al*, 2008) and have been implicated in the tumour responses to hypoxia (Royds *et al*, 1998; Karashima *et al*, 2003; Yokoi and Fidler, 2004). Previously, a positive correlation between HIF-1*α* and NF-*κ*B was shown in surgical colorectal cancer specimens (Kwon *et al*, 2010). In addition, *in vivo* animal and *in vitro* cell culture studies showed that HIF-1*α* activation was induced by NF-*κ*B in various cancer cells, including lung cancer cells, colon cancer cells, and osteosarcoma cells (Jung *et al*, 2003; Van Uden *et al*, 2008). However, these experiments were done mainly under normoxic conditions and little is known about this relationship in cancer cells under hypoxic conditions.

In gastric cancer, both HIF-1*α* (Zhong *et al*, 1999; Urano *et al*, 2006) and NF-*κ*B (Lee *et al*, 2005) have been reported to be overexpressed in surgical samples. Although HIF-1*α* was shown to increase angiogenesis and tumour growth of gastric cancer (Stoeltzing *et al*, 2004), the regulatory mechanism of HIF-1*α* activation in gastric cancer remains unclear. The present study investigated the correlation between HIF-1*α* and NF-*κ*B RelA in 251 surgically excised human gastric carcinoma tissues. In addition, we performed cell culture and animal studies after establishment of a stable gastric cancer cell line overexpressing supersuppressive mutant form of I*κ*B*α* (I*κ*B*α*M).

## Materials and methods

### Patients and tissue array methods

A total of 251 surgically resected human gastric cancer specimens were obtained from the Department of Pathology, Seoul National University College of Medicine from 1 January to 30 June, 1995, and six paraffin array blocks were prepared by Superbiochips Laboratories (Seoul, Korea), as previously described (Lee *et al*, 2003). Briefly, core tissue biopsies (2 mm in diameter) were taken from individual paraffin-embedded gastric tumours (donor blocks) and arranged in a new recipient paraffin block (tissue array block) using a trephine apparatus. As we have reported previously (Lee *et al*, 2001), the staining results of the different intratumoural areas of gastric carcinomas in these tissue array blocks showed an excellent agreement. A core was chosen from each case for the analysis. We defined an adequate case as a tumour occupying >10% of the core area. This protocol was reviewed and approved by the institutional review board of the Seoul National University (approval no. C-0603-162-170).

### Cell culture

A human gastric cancer cell line SNU-668 was obtained from the Korean Cell Line Bank (Seoul, Korea), cultured in RPMI-1640 medium (Life Technologies, Grand Island, NY, USA) containing 10% fetal bovine serum (Life Technologies), and maintained in a 37 °C humidified incubator containing 95% air and 5% CO_2_.

### Infection with retroviral vectors expressing I*κ*B*α* supersuppressor

The control retroviral vector MFG.EGFP.IRES.puro has been previously described (Oh *et al*, 2001). MFG.EGFP.IRES.puro and the retroviral vector MFG.I*κ*B*α*M.IRES.puro, which encodes an I*κ*B*α*M, were generated and infected into three gastric cancer cell lines, as described previously (Lee *et al*, 2005; Cho *et al*, 2008).

### *In vivo* tumourigenesis assay

Eight-week-old male athymic nude mice (*BALB/cSlc-nu*) were purchased from SLC Inc. (Hamamatsu, Shizuoka, Japan). All animal procedures were performed in accord with the procedures described in Workman *et al* (2010). Tumours were established by injecting 1 × 10^7^ gastric cancer cells (SNU-668^Vector^ or SNU-668^I*κ*B*α*M^ cells) in 100 *μ*l of Matrigel (provided by Professor Hynda K Kleinman, George Washington University, Washington DC, USA) subcutaneously (s.c.) into both flanks of each of ten mice. Tumours were measured on alternate days using a caliper, and the tumour volumes were calculated using the following formula: (length × width × height) × (*π*/6). After killing, tumour xenografts were removed and prepared for immunohistochemistry or immunoblotting.

### Tumour histology and immunohistochemistry

Tissue specimens were fixed with 10% neutral-buffered formalin, and 4 *μ*m paraffin sections were then prepared. One section was stained with haematoxylin and eosin (H&E) for histological assessment, and the other sections were immunostained using a streptavidin peroxidase procedure after microwave antigen retrieval. The primary antibodies were anti-NF-*κ*B p65 (1 : 50; Santa Cruz Biotechnology, Santa Cruz, CA, USA), anti-proliferating cell nuclear antigen (PCNA) (1 : 100; DAKO, Carpinteria, CA, USA), anti-cleaved caspase-3 (1 : 100; Cell Signaling Technology, Beverly, MA, USA), anti-CD31 (1 : 100; Santa Cruz Biotechnology), and anti-HIF-1*α* (1 : 50, provided by Dr Jong-Wan Park in Seoul National University, Seoul). Specimens were incubated with the biotinylated secondary antibody against the corresponding primary antibody and then with avidin–biotin–peroxidase complex (Vectastain Elite ABC kit, Vector Laboratories, Burlingame, CA, USA). Visualisation was performed using diaminobenzidine (DAB). All immunostained sections were then lightly counterstained with Mayer's haematoxylin. Throughout the above analysis, negative controls were prepared by omitting the primary antibody. For PCNA staining, we evaluated 500 cells and counted the cells with nuclear staining for each specimen. The proliferation index was defined as follows: proliferation index (%)=100 × PCNA-positive cells/total cells.

### Quantification of microvessel density in xenograft tumours

Microvessel densities (MVDs) were determined by light microscopy/optical image analysis after immunostaining xenograft tumour sections with anti-CD31 antibody as described previously (Stoeltzing *et al*, 2004). The three most highly vascularised areas in areas of tumours near the tumour–normal tissue interface were selected. Photographs of CD31-immunopositive vessels in tumour sections were taken under a light microscope, and the cross-sectional areas of CD31-immunopositive structures (i.e., vessel areas) were quantified by capturing images, converting them to greyscale, and analyzing CD31-stained areas using NIH Image Analysis software (version 1.62; National Institute of Health, Bethesda, MD, USA) after setting one consistent intensity threshold for all slides. Then, CD31-positive areas were expressed as pixels squared per high-power field and were measured for all tumours.

### Transient transfection and luciferase reporter assay

The NF-*κ*B-luciferase reporter plasmid (pNF-*κ*B-luciferase) (Stratagene, La Jolla, CA, USA) contains a 5 × NF-*κ*B response element fused to luciferase. To determine whether NF-*κ*B activity is controlled by hypoxia, SNU-668 cells were transiently co-transfected with 0.4 *μ*g of pNF-*κ*B-luciferase and 0.4 *μ*g of *β*-galactosidase vector, an internal control, using LipofectAMINE Plus (Life Technologies). At 24 h after transfection, SNU-668 cells were incubated under either normoxic or hypoxic conditions. Luciferase activity was measured on an AutoLumat LB 9505c luminometer (Berthold Analytical Instruments, Nashua, Germany) and normalised by *β*-galactosidase activity. Luciferase activities are presented as relative values *vs* the normoxic level of the empty vector control.

### Preparation of nuclear and cytoplasmic extracts

Cells were lysed in 100 *μ*l buffer A (10 mM l^−1^ Tris (pH 8.0), 60 mM l^−1^ NaCl, 1 mM l^−1^ EDTA, 1 mM l^−1^ DTT, 0.1% NP-40, and 1 mM l^−1^ phenylmethylsulphonyl fluoride), incubated on ice for 5 min, and centrifuged (pulsing for 5 s at 4 °C), and the cytoplasmic extracts obtained (the supernatant) were transferred to fresh tubes. Glycerol was then added to 20%, and the extracts were stored at −80 °C until required. The pelleted nuclei were immediately washed in 1 ml buffer A without NP-40, spun as described above, and resuspended in 50 *μ*l buffer B (200 mM l^−1^ HEPES (pH 7.9), 0.75 mM l^−1^ spermidine, 0.15 mM l^−1^ spermine, 0.2 mM l^−1^ EDTA, 2 mM l^−1^ EGTA, 2 mM l^−1^ DTT, 20% glycerol, 1 mM l^−1^ phenylmethylsulphonyl fluoride, and 0.4 M l^−1^ NaCl). They were then extracted on ice for 10 min with occasional vortexing and centrifuged, and the supernatant was collected as nuclear extract and stored at −80 °C until required.

### Lentivirus-mediated short hairpin RNA (shRNA) silencing of HIF-1*α*

Hypoxia-inducible factor-1*α* shRNA lentiviral particles and control shRNA lentiviral particles were purchased from Santa Cruz Biotechnology. The HIF-1*α* shRNA lentiviral particles is a pool of concentrated, transduction-ready viral particles containing three target-specific constructs that encode 19–25 nt (plus hairpin) shRNA designed to knock down gene expression. Construct in the control shRNA lentiviral particle encodes a scrambled shRNA sequence. The viral infection was performed by incubating SNU-668, SNU-216, and SNU-484 gastric cancer cells in the culture medium containing lentiviral particles for 12 h in the presence of 5 *μ*g ml^−1^ Polybrene (Santa Cruz Biotechnology). Pooled puromycin (2 *μ*g ml^−1^)-resistant cells were harvested and stored for further analysis.

### Protein isolation and western blotting

Cells were lysed with 1 × Laemmli lysis buffer (2.4 M glycerol, 0.14 M Tris (pH 6.8), 0.21 M SDS, and 0.3 mM bromophenol blue) and then boiled for 10 min. Protein contents were measured using BCA Protein Assay Reagent (Pierce, Rockford, IL, USA). Samples were diluted with 1 × lysis buffer containing 1.28 M *β*-mercaptoethanol, and equal amounts of protein were loaded onto 8–12% SDS–polyacrylamide gels. Proteins were electrophoretically transferred to PVDF or nitrocellulose membranes, and membranes were then blocked with 5% nonfat dry milk in PBS/Tween-20 (0.1%, vol/vol) at 4 °C overnight. They were then incubated with a primary antibody against I*κ*B*α* (1 : 1000; Cell Signaling Technology), NF-*κ*B/p65 (1 : 1000; Santa Cruz Biotechnology), HIF-1*α* (1 : 250; BD transduction Laboratories, San Diego, CA, USA), VEGF (1 : 1000; Santa Cruz Biotechnology), *β*-actin (1 : 5000; Sigma, St Louis, MO, USA), or transcription factor IIB (TFIIB) (1 : 250; BD Transduction Laboratories) for 3 h. They were then incubated with a corresponding secondary antibody, either horseradish peroxidase-conjugated anti-rabbit IgG (1 : 2000; Zymed, San Francisco, CA, USA) or anti-mouse IgG (1 : 2500; Santa Cruz Biotechnology) for 2 h, and enhanced chemiluminescence (ECL) (Amersham Biosciences, Arlington Heights, IL, USA) was used for the visualisation of the immunoreactive proteins. Equal loading of the protein was confirmed by *β*-actin (for cytoplasmic proteins) and TFIIB (for nuclear proteins).

### Semiquantitative reverse transcription–polymerase chain reaction (SQ RT–PCR)

To quantify mRNA levels, we used a highly sensitive, SQ RT–PCR method, as previously described (Chun *et al*, 2001). Total RNAs were isolated using TRIZOL reagent purchased from Invitrogen (Carlsbad, CA, USA), and 1 *μ*g of RNAs were reverse-transcribed at 48 °C for 30 min. Complementary DNAs were amplified over 18 PCR cycles (94 °C for 30 s, 52 °C for 30 s, and 70 °C for 30 s) in a reaction mixture containing 5 *μ*Ci (*α*-^32^P)dCTP (NEN, Boston, MA, USA). The resulting PCR fragments (5 *μ*l) were electrophoresed on a 2% agarose gel at 100 V in 1 × TAE, and the gels were dried and autoradiographed. Primer sequences were 5′-CCCCAGATTCAGGATCAGACA-3′ and 5′-CCATCATGTTCCATTTTTCGC-3′ for HIF-1*α*, 5′-GGTGAAGTTCATGGATGTCT-3′ and 5′-TCTGCATTCACATTTGTTGT-3′ for VEGF, and 5′-ACACCTTCTACAATGAGCTG-3′ and 5′-CATGATGGAGTTGAAGGTAG-3′ for *β*-actin.

### Assessment of cell viability

SNU-668, SNU-484, and SNU-216 gastric cancer cells (2.5 × 10^4^ cells) were seeded into each well of 24-well plates and were allowed to grow under hypoxic conditions for 0–72 h. Cell numbers were then measured indirectly using the method reported by Kim *et al* (Hur *et al*, 2003). Briefly, cells were stained with 0.2% crystal violet aqueous solution in 20% methanol for 10 min, dissolved in 10% SDS, transferred into 96-well plates, and the absorbance was measured at 570 nm using an ELISA reader (Bio-Rad, Hercules, CA, USA).

### Statistical analysis

For tissue array analysis, statistical analyses were conducted using SPSS Version 11.0 statistical software program (SPSS, Chicago, IL, USA), and the *χ*^2^-test was used to determine the correlation between the nuclear expressions of NF-*κ*B and HIF-1*α*. For the animal and cell experiments, data were analyzed using SAS software (version 8.1; SAS Institute Inc., Cary, NC, USA), and the two-tailed Student's *t-*test was used to determine the significances of the results. The *P-*values of <0.05 were considered significant for all statistical analyses.

## Results

### HIF-1*α* activation in relation to NF-*κ*B activation in clinical gastric cancer samples

**Figure 1:**
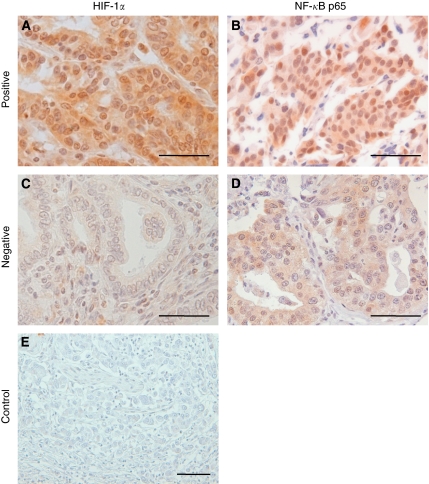
Representative immunohistochemical features of HIF-1*α* and NF-*κ*B in human gastric cancer specimens. Positive (**A** and **B**) *vs* negative (**C** and **D**) examples of gastric cancer for HIF-1*α* (**A** and **C**) and NF-*κ*B (**B** and **D**). (**E**) A negative control of gastric cancer specimen treated without primary antibodies. Scale bars=50 *μ*m.

To confirm the correlation between HIF-1*α* and NF-*κ*B in human gastric cancer tissues, we performed immunohistochemistry on tissue array slides containing 251 human gastric cancer specimens. Figure 1 shows that both HIF-1*α* (Figure 1A) and NF-*κ*B (Figure 1B) are expressed in both the nucleus and the cytoplasm of tumour cells. Cells showing distinct nuclear staining, regardless of the presence of cytoplasmic staining, were considered to express activated HIF-1*α* or NF-*κ*B. Immunostaining results were considered to be positive when ⩾5% (for HIF-1*α*) and ⩾10% (for NF-*κ*B) of tumour cell nuclei were stained (Figure 1A and B, respectively). Positive immunoreactivity for nuclear HIF-1*α* was found in 69 of 251 (27%) gastric cancer specimens. In addition, nuclear NF-*κ*B was found in 46 of 251 (18%) of gastric cancer specimens. Data concerning the correlation between the activations of HIF-1*α* and NF-*κ*B are summarised in Table 1. Nuclear HIF-1*α* expression was found to be significantly and positively correlated with nuclear NF-*κ*B expression (*P*<0.001).

### Effect of NF-*κ*B inhibition on tumour growth and MVD in the nude mouse xenograft model

**Figure 2:**
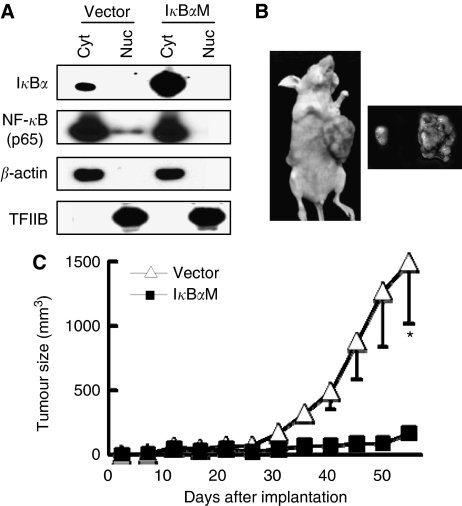
Effect of NF-*κ*B inhibition on tumour growth in a xenograft tumour model. Each mouse was ectopically implanted with gastric cancer cells mixed in Matrigel on both flanks. (**A**) SNU-668 cells were transduced with the MEG.I*κ*B*α*M.IRES.puro (I*κ*B*α*M) retroviral vector or MEG.EGFP.IRES.puro as a control empty vector. Immunoblotting for I*κ*B*α* or NF-*κ*B protein was performed using an anti-I*κ*B*α*- or an anti-NF-*κ*B p65 antibody, respectively. *β*-Actin or transcription factor IIB (TFIIB) was used as a control for cytoplasmic (Cyt) or nuclear (Nuc) protein, respectively. Immunoreactive proteins were visualised by enhanced chemiluminescence. (**B**) A representative nude mouse bearing xenograft tumours derived from SNU-668^I*κ*B*α*M^ cells (right flank) and SNU-668^Vector^ cells (left flank). Photographs were taken 54 days after s.c. injection. (**C**) Tumour size was measured over time. Values represent the means±s.d. (*n*=7) of tumour size and were analyzed using the Student’s *t-*test. ^*^P<0.05 *vs* SNU-668^Vector^.

In order to confirm the correlation between HIF-1*α* and NF-*κ*B observed in the clinical gastric cancer samples, we produced stable SNU-668 cell lines overexpressing either empty vector (SNU-668^Vector^) or I*κ*B*α*M (SNU-668^I*κ*B*α*M^). Western blotting showed that I*κ*B*α*M overexpression inhibited the nuclear translocation of NF-*κ*B p65 in SNU-668 cells (Figure 2A).

We then established gastric carcinoma xenografts derived from these cells and examined the effects of NF-*κ*B p65 inhibition on tumour growth. We found that 7 out of 10 mice showed tumour formation (Figure 2B). On the final day of measurement (day 54), SNU-668^I*κ*B*α*M^ tumours (mean=170.0 mm^3^) were significantly smaller than SNU-668^Vector^ tumours (mean=1490.6 mm^3^) (*P*=0.035; Figure 2C). These results indicated that NF-*κ*B inhibition suppresses the gastric tumour growth.

**Figure 3:**
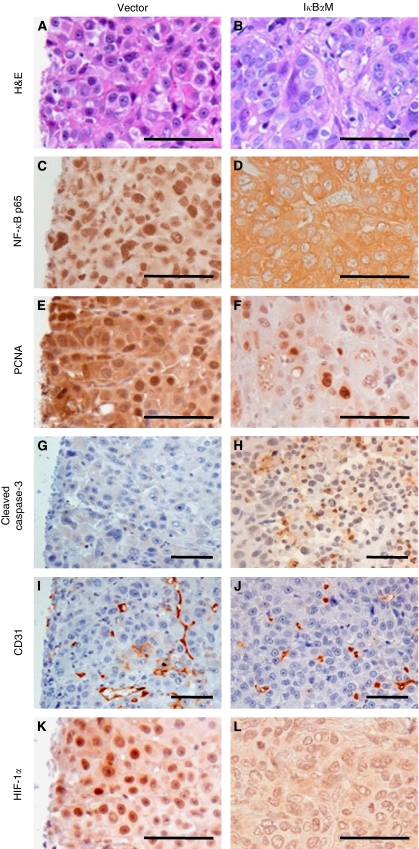
Representative immunohistochemical findings of the effect of I*κ*B*α*M overexpression in differently sized xenograft tumours derived from SNU-668^Vector^ cells (left lanes) or SNU-668^I*κ*B*α*M^ cells (right lanes). Tumour sections were stained with H&E (**A** and **B**), anti-NF-*κ*B p65 (**C** and **D**), anti-PCNA (**E** and **F**), anti-cleaved caspase-3 (**G** and **H**), anti-CD31 (**I** and **J**), or anti-HIF-1*α* (**K** and **L**). All sections were lightly counterstained with haematoxylin. Scale bars=50 *μ*m.

**Figure 4:**
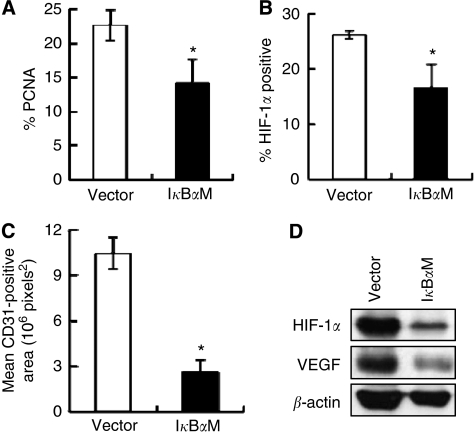
Effects of I*κ*B*α*M overexpression on gastric cancer xenografts. (**A**–**C**) The frequencies of immunopositive cells for PCNA (**A**) or HIF-1*α* (**B**) and the areas of blood vessels immunostained for CD31 (**C**) in differently sized gastric cancer xenografts (*n*=7) were quantified. Values represent means±s.d. and were determined using the Student's *t-*test. ^*^P<0.05 *vs* SNU-668^Vector^. (**D**) Immunoblotting analysis of HIF-1*α* expression in similarly sized xenograft tumours.

To investigate the mechanism by which NF-*κ*B promotes gastric tumour growth, we performed immunohistochemistry on xenograft tissue sections. First, we identified that nuclear NF-*κ*B p65 expression was less frequent in SNU-668^I*κ*B*α*M^ tumours than in SNU-668^Vector^ tumours (Figure 3C and D), indicating that NF-*κ*B activation was inhibited by I*κ*B*α*M overexpression in tumours. Next, the protein expressions of PCNA, a proliferation-related marker, and cleaved caspase-3, an apoptosis-related marker, were analyzed. The immunoreactivity of PCNA was found less frequently in the nuclei of SNU-668^I*κ*B*α*M^ tumour cells than in those of SNU-668^Vector^ tumour cells (*P*=0.012; Figures 3E and F and 4A). In addition, the immunoreactivity of cleaved caspase-3 was enhanced in SNU-668^I*κ*B*α*M^ tumour cells compared with in SNU-668^Vector^ tumour cells (Figure 3G and H). Thus, the NF-*κ*B activity in the gastric cancer xenograft appears to be correlated positively with cell proliferation, and negatively with apoptosis.

We then examined whether NF-*κ*B activity was associated with tumour angiogenesis. Immunohistochemical staining of an endothelial cell marker CD31 was performed on tissue sections of xenografted tumours (Figure 3I and J). Optical image analyses showed that the CD31-positive vessel area was noticeably smaller in SNU-668^I*κ*B*α*M^ tumours (mean=2.6 × 10^6^ pixels^2^) than in SNU-668^Vector^ tumours (mean=10.4 × 10^6^ pixels^2^) (*P*<0.001; Figure 4C). Thus, the NF-*κ*B p65 activity is likely to be positively correlated with angiogenesis in gastric tumours.

### Effect of NF-*κ*B inhibition on HIF-1*α* and VEGF expression

Hypoxia-inducible factor-1*α* was previously found to increase angiogenesis and tumour growth in gastric cancer (Stoeltzing *et al*, 2004). As the present study showed that I*κ*B*α*M overexpression suppressed MVD in xenografted tumours derived from SNU-668 cells, we assessed whether HIF-1*α* mediates the effect of NF-*κ*B activation in gastric cancer. Immunohistochemistry showed that the frequency of HIF-1*α*-positive cells was significantly lower in SNU-668^I*κ*B*α*M^ tumours (mean=16.6%) than in SNU-668^Vector^ tumours (mean=26.2% *P*=0.001; Figures 3K, L and 4B). Thus, these results indicate that HIF-1*α* is a downstream molecule of NF-*κ*B.

Furthermore, to exclude the possibility that this difference in HIF-1*α* immunoreactivity is correlated with the size of xenograft tumours rather than NF-*κ*B activity, we compared HIF-1*α* expression in three sets of similarly sized xenograft tumours: the sizes of SNU-668^I*κ*B*α*M^ tumours were 251.2, 255.2, and 255.8 mm^3^, and the sizes of corresponding vector control tumours were 255.4, 256.3, and 259.5 mm^3^. Western blot analysis showed that I*κ*B*α*M overexpression decreased the expressions of HIF-1*α* and VEGF in similar sized tumours (Figure 4D).

**Figure 5:**
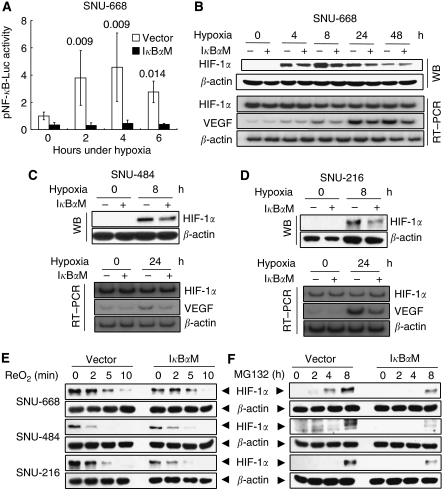
Effect of NF-*κ*B inhibition on the expressions of HIF-1*α* and VEGF in gastric cancer cell lines *in vitro*. (**A**) NF-*κ*B luciferase reporter assay showed that hypoxia enhanced NF-*κ*B activation in SNU-668^Vector^ cells, but not in SNU-668^I*κ*B*α*M^ cells. Values represent means±s.d. of three separate experiments and were determined using the Student's *t-*test. *P vs* normoxic control. (**B**–**D**) Protein and mRNA expressions of HIF-1*α* and *β*-actin were determined by western blotting (WB) and SQ RT–PCR (RT–PCR), respectively, after cells were cultured under hypoxic conditions for the indicated period. (**E**) Oxygen-dependent degradation of HIF-1*α*. Cells were cultured to reach 60% confluence, and were preincubated under hypoxic conditions for 8 h. Then, at the indicated time of re-oxygenation, HIF-1*α* levels were analyzed by western blotting. (**F**) HIF-1*α* protein synthesis. After pretreatment with cycloheximide (100 *μ*M) for 1 h, cells were incubated with MG132 (10 *μ*M) to prevent the degradation of newly synthesised HIF-1*α*. The HIF-1*α* protein levels were determined at the indicated time by western blotting.

As hypoxia is an important environment for tumour growth and progression, we performed *in vitro* cell culture experiments and investigated the correlation between hypoxia and the NF-*κ*B activation in gastric cancer cells. Luciferase reporter assay showed that hypoxia induced NF-*κ*B activation in SNU-668^Vector^ cells after 2 h of hypoxia (*P*=0.009 *vs* normoxic control), and that basal NF-*κ*B activity was essential for hypoxia-induced NF-*κ*B activation in gastric cancer cells (Figure 5A). In addition, I*κ*B*α*M overexpression reduced HIF-1*α* protein expression under hypoxic conditions (Figure 5B, top). However, SQ RT–PCR showed that I*κ*B*α*M overexpression did not alter the expression of HIF-1*α* mRNA, although it decreased the expression of hypoxia-induced VEGF mRNA in SNU-668^I*κ*B*α*M^ cells (Figure 5B, bottom). Consistent results were shown in other gastric cell lines SNU-484 (Figure 5C) and SNU-216 (Figure 5D).

### Effects of I*κ*B*α*M overexpression on the degradation and *de novo* synthesis of HIF-1*α* protein

To obtain a better understanding of the mechanism involved in NF-*κ*B-induced HIF-1*α* activation in gastric cancer cells, we investigated whether I*κ*B*α*M stimulates HIF-1*α* degradation or inhibits its synthesis. Using three gastric cancer cell lines, we first analyzed the effect of NF-*κ*B on the oxygen-dependent degradation of HIF-1*α* protein. Hypoxia-inducible factor-1*α* was first stabilised by exposure to hypoxia, and then destabilised by re-oxygenation. We found that I*κ*B*α*M overexpression did not affect the oxygen-dependent degradation rate of HIF-1*α* protein (Figure 5E). Next, we checked whether I*κ*B*α*M overexpression inhibits the synthesis of HIF-1*α* protein. Cells were pretreated with cycloheximide to remove the remaining HIF-1*α*, and further degradation of HIF-1*α* protein was blocked using a proteasome inhibitor MG132. We then examined the time course of HIF-1*α* accumulation. Figure 5F shows that HIF-1*α* protein was substantially synthesised after 4 h and that the *de novo* synthesis of HIF-1*α* was impaired by I*κ*B*α*M overexpression. These results indicate that I*κ*B*α*M overexpression inhibits synthesis, not stabilisation, of HIF-1*α* protein. Thus, the hypoxic activation of NF-*κ*B seems to contribute to the expression of HIF-1*α* protein at the translational level.

### Effect of HIF-1*α* shRNA expression on gastric cancer cell viability *in vitro* under hypoxic conditions

**Figure 6:**
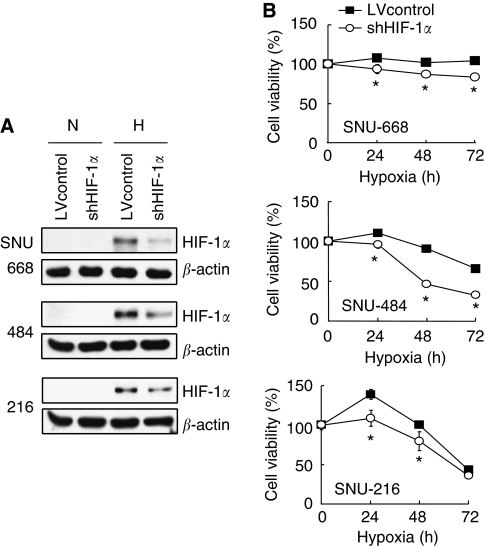
Effect of HIF-1*α* suppression induced by lentivirus-delivered shRNA on the cell viability of gastric cancer cell lines SNU-668 (top), SNU-484 (middle), and SNU-216 (bottom). Cells were infected with a lentivirus containing a construct, which encodes either HIF-1*α* shRNA (denoted as shHIF-1*α*) or scrambled shRNA (denoted as LVcontrol). Expression levels of HIF-1*α* and *β*-actin proteins were determined by western blotting after cells were exposed to normoxia (N) or hypoxia (H) for 8 h (**A**). Cell viability was measured at the indicated time of hypoxia exposure and was represented as the percentage of cell number expressing HIF-1*α* shRNA *vs* vector control cells (**B**). Values represent the means±s.d. (*n*=4). *P<0.05.

To investigate the role of HIF-1*α* in gastric tumour growth derived from SNU gastric cancer cells, we blocked the HIF-1*α* pathway in three gastric cancer cell lines using shRNA expression. Our results showed that shRNA-mediated downregulation of HIF-1*α* expression reduced the cell viability of SNU-668, SNU-484, and SNU-216 gastric cancer cells *in vitro* under hypoxic conditions (Figure 6). These data are consistent with a previous *in vivo* findings reported by Stoeltzing *et al* (2004), who showed that xenografted tumours derived from human gastric cancer TMK-1 cells overexpressing dominant-negative-HIF-1*α* had smaller volumes than those derived from vector control cells.

## Discussion

Gastric cancer is one of the most common malignancies worldwide and the leading cause of cancer-related motility in Asian countries (Parkin *et al*, 1993). Although angiogenesis is an important aspect of tumour growth and progression and is considered the most important predictor of overall survival in gastric cancer (Gong *et al*, 2005), little is known about the molecular events critical to the gastric cancer angiogenesis.

Previously, it was found that HIF-1*α* activation was significantly correlated with VEGF protein expression, and this overexpression was a prognostic factor in patients with gastric cancer (Mizokami *et al*, 2006; Urano *et al*, 2006). Although it was shown that HIF-1*α* inhibition reduced angiogenesis and tumour growth in xenografted gastric tumours (Stoeltzing *et al*, 2004), it has become evident that the mechanism underlying the activation of HIF-1 in various cancer cells depends on cancer type (Li *et al*, 2005). In the present study, NF-*κ*B inhibition suppressed angiogenesis, as was manifested by decreased MVD, and tumour growth of gastric cancer xenografts. Consistently, HIF-1*α* shRNA expression decreased the cell growth of gastric cancer cell lines. In addition, immunohistochemical tissue array analysis showed that HIF-1*α* was constitutively expressed in 27% of the 251 surgical samples of gastric carcinomas, and that this was positively correlated with NF-*κ*B activation (*P*<0.001). Thus, we investigated whether HIF-1*α* mediates the role of NF-*κ*B in gastric cancer angiogenesis.

In xenografted gastric tumours, we used stable gastric cancer cells infected with a retrovirus overexpressing I*κ*B*α*M, which could prove to be a powerful tool to obtain further insight into the role of NF-*κ*B in the regulation of HIF-1 activity. We found that I*κ*B*α*M overexpression decreased HIF-1*α* protein expression as well as MVD in xenograft tumours. In addition, I*κ*B*α*M overexpression decreased VEGF mRNA expression in cell culture experiments. Thus, these results suggest that HIF-1*α* is a downstream molecule of NF-*κ*B in the angiogenesis pathway in gastric cancer.

Hypoxia-inducible factor-1 activation was shown to be induced by NF-*κ*B in lung cancer cells and colon cancer cells under normoxic conditions (Jung *et al*, 2003; Van Uden *et al*, 2008). In contrast, HIF-1 activation by NF-*κ*B was hypoxia dependent in smooth muscle cells and embryonic kidney cells (Belaiba *et al*, 2007; Van Uden *et al*, 2008). In the present study, through a cell culture system, we found that hypoxia was involved in the NF-*κ*B effect on the expressions of HIF-1*α* protein and VEGF mRNA in gastric cancer cells. Thus, the NF-*κ*B/HIF-1/VEGF pathway in gastric cancer cells might be activated under hypoxic conditions.

In the present study, cell culture experiments showed that NF-*κ*B inhibition suppressed hypoxia-induced HIF-1*α* protein expression, but not HIF-1*α* mRNA expression. Furthermore, we found that I*κ*B*α*M overexpression inhibited synthesis, not degradation, of HIF-1*α* protein. Thus, the hypoxic activation of NF-*κ*B seems to contribute to the accumulation of HIF-1*α* protein at the translational level, but not at the transcriptional or post-translational level. These results are not in accord with earlier findings shown in normal cells such as smooth muscle cells (Belaiba *et al*, 2007) and embryonic kidney cells (Van Uden *et al*, 2008), which suggested that NF-*κ*B targets and transactivates the *HIF-1α* gene. This discrepancy may come from the different cell types (cancer cell *vs* non-cancer cells), as the mechanism underlying the activation of HIF-1*α* is specifically tailored according to cell type (Li *et al*, 2005).

In conclusion, the results obtained from human gastric cancer specimens, gastric tumour xenografts, and cell culture experiments indicate that HIF-1*α* mediates NF-*κ*B-induced angiogenesis by increasing VEGF expression and MVD, and that this occurs under hypoxic conditions. Thus, HIF-1*α* and NF-*κ*B may be candidate molecular targets for gastric cancer therapy, and in particular, blocking the NF-*κ*B/HIF-1*α* pathway with appropriate inhibitors might be useful therapeutically for treating gastric carcinoma.

## Figures and Tables

**Table 1:** Correlation between the nuclear expressions of HIF-1*α* and NF-*κ*B in human gastric carcinomas

	**HIF-1*α* (nuclear)**	**HIF-1*α* (negative and cytoplasm)**	
	***n* (%)**	***n* (%)**	***P-*value**
*NF-κB*			
Nuclear	24 (52)	22 (48)	<0.001^a^
Negative and cytoplasm	45 (22)	160 (78)	

Abbreviations: HIF-1=hypoxia-inducible factor-1; NF-*κ*B=nuclear factor-*κ*B.

a Considered to be statistically significant.
